# Prevalence of Multidrug-Resistant *Klebsiella pneumoniae* Clinical Isolates in Nepal

**DOI:** 10.1155/2022/5309350

**Published:** 2022-02-22

**Authors:** Ranjeeta Odari, Prabin Dawadi

**Affiliations:** Nepal Academy of Science and Technology, Khumaltar, Lalitpur, Nepal

## Abstract

**Background:**

Multidrug-resistant *Klebsiella pneumoniae* (MDR-KP) are becoming increasingly common over the world. The focus of this research was to get a quantitative assessment of *K*. *pneumoniae* and their multidrug resistance (MDR) profile in Nepal.

**Methods:**

Three electronic databases: PubMed, Google Scholar, and Research4Life were used to search publications specifying *K*. *pneumoniae* infections and/or their MDR status from January 2015 to October 2021. Preferred Reporting Items for Systematic Reviews and Meta-Analysis (PRISMA) guidelines was followed for the review, and R language 4.1.1 was used for analysis. Depending upon heterogeneity of data, we used random model for pooled data to examine the prevalence of the organism and the multidrug resistance.

**Results:**

Evaluation included 16 studies, and the pooled estimation of *K*. *pneumoniae* in total clinical samples was 3% (95% CI; 0.01–0.05). In the meta-analysis, 14 studies were combined for determining the prevalence of *K*. *pneumoniae* in total positive clinical isolates which was 16% (95% CI: 0.11–0.20), while from 12 research studies, MDR status in the pathogen was found to be 64% (95% CI, 0.53–0.74).

**Conclusion:**

The MDR status of *K*. *pneumoniae* as well as the prevalence of the bacteria in Nepal was analyzed which showed alarming situation about administration of antibiotics and indicated choosing and developing reliable antibiotic strategies.

## 1. Introduction

*Klebsiella pneumoniae* is a Gram-negative bacteria that can be frequently found in the mouth, on the skin, and in the intestines, as well as in natural environments like water and soil [1–3]. The organism is one of the most common opportunistic bacteria linked to nosocomial and community-acquired infections, especially in immune-compromised patients responsible for causing infections in the urinary tract, respiratory tract, lower biliary duct, soft tissue, blood, surgical wounds, and liver [4–11]. *K*. *pneumoniae* has emerged as a major clinical and public health problem due to the rising prevalence of the infections caused by emerging multidrug-resistant strains [6, 7, 12].

The therapeutic options for infections caused by multidrug-resistant (MDR) *K*. *pneumoniae* are often limited. The prevalence of multidrug resistance bacterial species has risen significantly since the introduction and widespread use of new generation extended range antibiotics. By manufacturing enzymes like extended spectrum-lactamase (ESBLs), carbapenemase, and forming biofilms, the organism has been reported to develop antibiotic resistance faster than other bacteria [12, 13]. One of the primary causes in the production and spread of highly resistant bacteria for health-care-associated disorders is the intensive and continuous use of antibiotics in the hospital context [14]. The bacterium is resistant to a wide spectrum of medications, including fluoroquinolones and aminoglycosides [15–17]. As a result of increased resistance, choosing an effective antibiotic treatment for hospital-acquired infections is becoming increasingly difficult around the world [18].

Drug resistance in developing countries like Nepal have several reasons, including health-care professionals' behaviors and patients' attitudes toward antibiotic use, as well as antimicrobial supply networks in the population. This is the first meta-analysis so far according to our knowledge emphasizing in prevalence of *K*. *pneumoniae* infections and their multidrug status in Nepal. As a reason, the objective of this research was to explore at those characteristics in the organism isolated from Nepal in order to provide situation of the concerns. This analysis could create a deeper understanding about persistence of the infection and their MDR profile alerting the authorities locally and globally.

## 2. Methods

Following the PRISMA guidelines, this review was conducted using Medline/PubMed, Research4Life, and Google Scholar [19]. The terms used in the search were “MDR *K. pneumoniae* in Nepal” and “*K. pneumoniae* in Nepal.” The searches were restricted to articles published between 2015 and 2021, with work dates ranging from January 1, 2015, and October 20, 2021.

### 2.1. Eligibility Criteria

Each study's eligibility was chosen separately after the search results were examined, and any disagreements were resolved through discussion among the authors. Any discrepancies that arose during the review over whole articles were resolved by a majority vote. The title and abstract were used to evaluate the results of the initial search procedure. For inclusion and exclusion criteria, the whole texts of relevant papers were assessed.

### 2.2. Inclusion Criteria

Observational studies from Nepal that recorded the occurrence of *K*. *pneumoniae* in humans and/or their multidrug resistance profile were selected for quantitative synthesis. We considered all standard guidelines for antimicrobial therapy but only Clinical and Laboratory Standards Institute (CLSI) guidelines were found to be used in the included studies. Standard laboratory method included Kirby–Bauer disk diffusion method for antibiotic susceptibility test in all studies. In our study, MDR was defined as the organisms resistant to at least one antimicrobial agent in three or more antimicrobial categories.

### 2.3. Exclusion Criteria

The articles that reported the pathogen from samples other than human samples were excluded to minimize heterogeneity and bias. Articles that did not apply established procedures for detecting drug resistance (according to guidelines), did not provide the sample size or had inappropriate data, were also eliminated.

### 2.4. Data Synthesis and Analysis

The screened publications contained variables like first author, publication date, study site, sample size, *K*. *pneumoniae*, and MDR-*K*. *pneumoniae* (supplementary file 1 (available here)). Statistical studies were performed using the R programming language (Meta package). Percentages were used to represent the distributions of category variables. The incidence of bacteria and the drug resistance in clinical settings was estimated as a proportion with a 95% confidence interval and shown as a forest plot using the random effects model. To identify study heterogeneity, the Cochran *Q* test was utilized, with a *p* value of less than 0.10 indicating significant heterogeneity. The *I*^2^ statistic was used to measure how much heterogeneity contributed to the total variation in research estimates. *I*^2^ values of 25%, 25–75%, and >75% indicate low, moderate, and high heterogeneity, respectively [20].

## 3. Results

### 3.1. Summary of Selected Study

**Figure 1:**
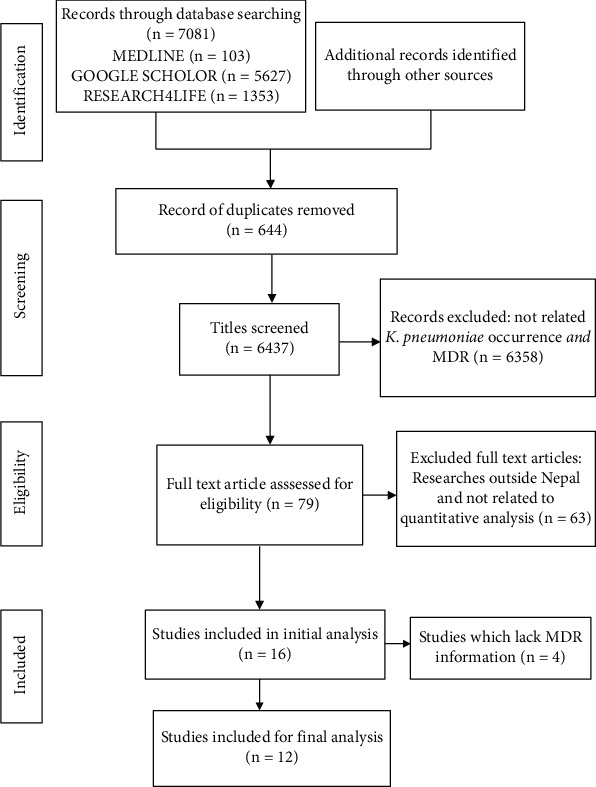
A flow diagram of the search strategy according to the Preferred Reporting Items for Systematic Reviews and Meta-Analyses (PRISMA) guidelines.

The search approach yielded a total of 7081 important potential articles. After 644 duplicates were removed, the remaining 6437 papers were screened again by title and abstract, with 79 being chosen for full-text examination. A total of 16 papers were included in the quantitative meta-analysis for determining the prevalence of the pathogen, while only 12 papers were included for analyzing their MDR profile. In searching the relevant information, 63 articles were excluded due to a lack of complete information on MDR and the presence of the target organism, as well as investigations conducted outside Nepal. Among included studies, a majority of the investigations were from tertiary care hospitals in Kathmandu valley. In the specified work duration, total samples from all included studies were 29,741 in which 4099 showed positive growth upon culture. Among those positive isolates, 643 isolates were *K*. *pneumoniae* and 327 were determined to be MDR-KP. The samples were mostly from children and adults. Most of the patients were from the general section, while a study reported patients from ventilation. Patients suffering from UTI and neonatal sepsis were also included in this study. The flowchart for study selection is shown in Figure 1.

### 3.2. Meta-Analysis on Prevalence of *K*. *pneumoniae*

**Figure 2:**
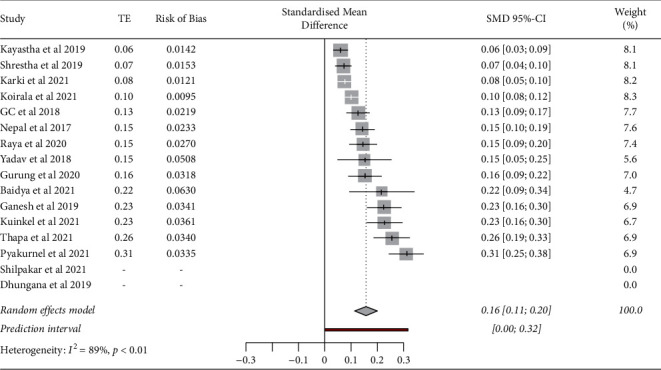
Prevalence of *K*. *pneumoniae* from clinical positive isolates in Nepal from 14 different studies ((*I*^2^ = 89%, pooled prevalence = 16%, 95% CI: 0.11–0.20, *p* < 0.01).

The pooled estimation of *K*. *pneumoniae* in clinical settings in various processed samples (29,741) that came in laboratory for investigations from 16 papers was 3% (95% CI; 0.01–0.05), with significant heterogeneity among studies (*p* < 0.01; *I*^2^ = 94%) (supplementary file 1 (available here)). The pooled estimation of the prevalence of the bacteria among the total positive isolates (4099) from 14 studies was 16% (95% CI; 0.11–0.20) with high heterogeneity (*p* < 0.01, *I*^2^ = 89%) (Figure 2).

### 3.3. Multidrug Resistance

**Figure 3:**
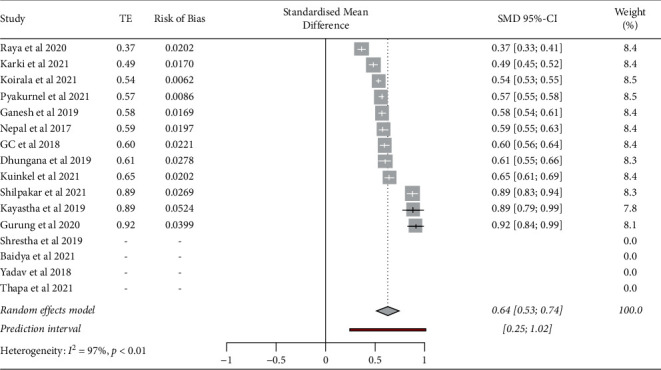
Prevalence of MDR-*K*. *pneumoniae* from clinical isolates in Nepal from 12 different studies (*I*^2^ = 97%, pooled prevalence = 64%, 95% CI: 0.53–0.74, *p* < 0.01).

The pooled prevalence of multidrug resistance in total 643 *K*. *pneumoniae* from 12 researches was 64% (95% CI, 0.53–0.74). There was high heterogeneity among analyzed studies (*p* < 0.01, *I*^2^ = 97%) (Figure 3).

## 4. Discussion

The prevalence of *K*. *pneumoniae* infections isolated from humans and their MDR status 2015 to 2021 were assessed in this meta-analysis. This is the first comprehensive meta-analysis on the prevalence of the pathogen in Nepal. We hope the information acquired thus far will help provide background aspects of drug resistance in order to avoid pan drug resistance in humans. The number of studies included in the meta-analysis was limited due to the study's search limitations. In Nepal, the occurrence of the pathogen to cause the infection ranges from about 7% to 37% displaying rise in the infection [21–36]. The results in this study determined the combined estimation of *K*. *pneumoniae* isolates in both overall suspected samples (3%) and positive isolates in the country (16%). In this investigation, a significant multidrug prevalence was found among *K*. *pneumoniae* (pooled prevalence, 64%). In various locations, the multidrug resistance was seen to vary from 6% to 91% [4, 23, 26, 27, 29–36].

Urinary tract infections, respiratory tract infections, and septicemia may be caused by *K*. *pneumoniae*, especially in immunocompromised people [8, 14, 37]. However, treatment options for infections caused by multidrug-resistant *K*. *pneumoniae* are frequently restricted [38]. Since the introduction, followed by unrestricted usage of new generation extended range antibiotics, the prevalence of multidrug resistant bacteria has increased substantially [39]. As a result of this resistance, there is a rising global difficulty with choosing an effective antibiotic treatment for hospital-acquired infections [8, 14, 18]. The pathogen is also involved in the transmission of antibiotic-resistant genes from bacteria in the environment to clinically significant pathogens [10, 40].

The meta-analysis has been important to determine the pathogen's pooled estimate and resistance to more than two classes of antibiotics. The antibiotic selective pressure in pathogen may develop the multiresistance to antibiotics, and to date, some strains of the organism have developed resistance to almost all currently available antimicrobial agents, including carbapenems, which were previously thought to be the drugs of choice for treating infection by this microorganism [31]. MDR infections are most common in complicated patients who require long-term antibiotic therapy and hospitalization and who frequently undergo invasive procedures [4, 5, 34, 38, 41]. Antimicrobial resistance risk factors may differ based on the type of organism and population analyzed [42].

Due to the small number of reports, the current study had limitations. Many of the investigations were excluded from the study due to a lack of relevant information. Out of 16 papers, only 12 studies were eligible to determine the status of multidrug resistance in the pathogen. In the meta-analysis, another limitation was the type of patient was not considered. More research into the prevalence of pathogens as well as multidrug resistance patterns in clinical settings based on infection site and patient type is required. Such research could provide a more comprehensive picture of MDR patterns in clinical settings, as well as help in controlling those resistant bacteria that have a high risk of disease development.

## 5. Conclusion and Recommendation

In Nepal, the prevalence of *K*. *pneumoniae* has been shown to be high (16%), with MDR patterns in the pathogen reaching up to 64%. This is a concerning scenario, and relevant authorities must remain vigilant in order to prevent worsening of the situation. This study suggests that more research into the process and reasons of antibiotic resistance in the organism, as well as the development of new antibiotics, is required. Therefore, antimicrobials used in treatment should be carefully managed.

## Data Availability

The data used to support the finding of this study are available from the corresponding author upon request.

## Supplementary Materials

Supplementary Materials Data on studies, occurrence of *K*. *pneumoniae*, MDR pattern, and publication bias are present in supplementary file 1.
