# Supplementary material for: Prevalence of Multidrug-Resistant Klebsiella pneumoniae Clinical Isolates in Nepal
Source: J Trop Med. 2022 Feb 22;2022:5309350. doi: 10.1155/2022/5309350 (PMC8888090; doi:10.1155/2022/5309350)
Supplement: Supplementary Materials — Data on studies, occurrence of K. pneumoniae, MDR pattern, and publication bias are present in supplementary file 1. [file 5309350.f1.docx]

| Study | Work duration | Total Samples | Total positive  isolates | *K*. *pneumoniae* | MDR |
| --- | --- | --- | --- | --- | --- |
| (1) | June 2015 to January 2016 | 1599 | 197 | 45 | 26 |
| (2) | January to June 2018) | 1962 | 314 | 23 |  |
| (3) | April 2017–April 2018 | 5545 | 203 | 30 | 11 |
| (4) | June 2018 to January 2019 | 720 | 280 | 88 | 50 |
| (5) | July 2019 to January 2020 | 70 | 55 | 12 |  |
| (6) | April and September 2019 | 5690 | 1142 | 118 | 64 |
| (7) | April and September 2018 | 1013 | 154 | 24 | 22 |
| (8) | March to August, 2019 | 3216 | 529 | 41 | 20 |
| (9) | May 2015 to December 2015 | 1568 | 268 | 39 | 23 |
| (10) | June 2018 to November 2018. | 770 |  | 35 | 31 |
| (11) | April to September 2015 | 350 | 59 | 9 |  |
| (12) | July 2018 and January 2019 | 585 | 224 | 58 |  |
| (13) | August 2017 to January 2018 | 1453 | 299 | 18 | 16 |
| (14) | January 2016 to June 2016 | 1500 |  | 28 | 17 |
| (15) | November, 2016 – April, 2017 | 1503 | 270 | 35 | 21 |
| (16) | February and August 2019 | 2197 | 175 | 40 | 26 |
| Total |  | 29,741 | 4099 | 643 | 327 |

**Prevalence of multi-drug resistant *Klebsiella* *pneumoniae* clinical isolates in Nepal**

Ranjeeta Odari^1^, Prabin Dawadi*^1^

^1^Nepal Academy of Science and Technology, Khumaltar, Lalitpur, Nepal.

Correspondence: Prabin Dawadi. Nepal Academy of Science and Technology, Khumalatar,

Table 1: Occurrence of *K*. *pneumoniae* in clinical isolates and their MDR status

Figure 1: Prevalence of *K*. *pneumoniae* in total clinical samples


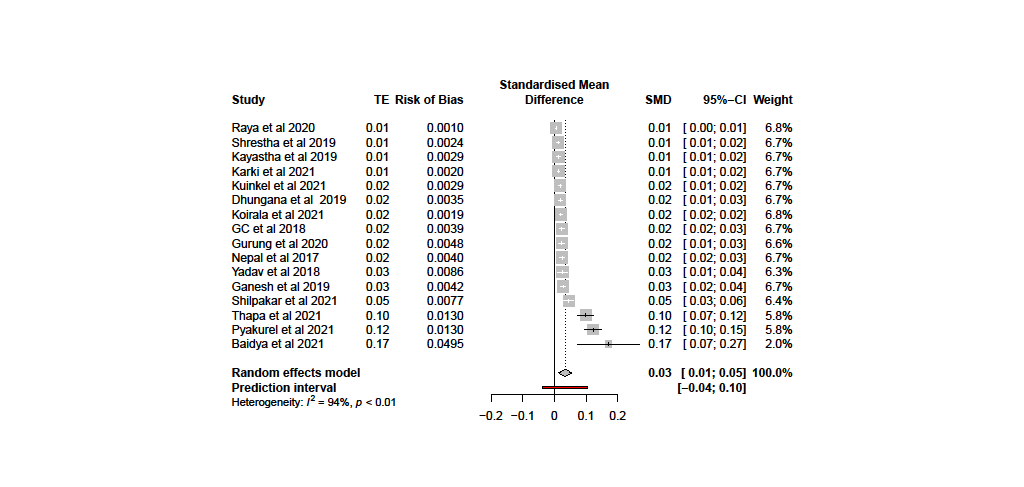

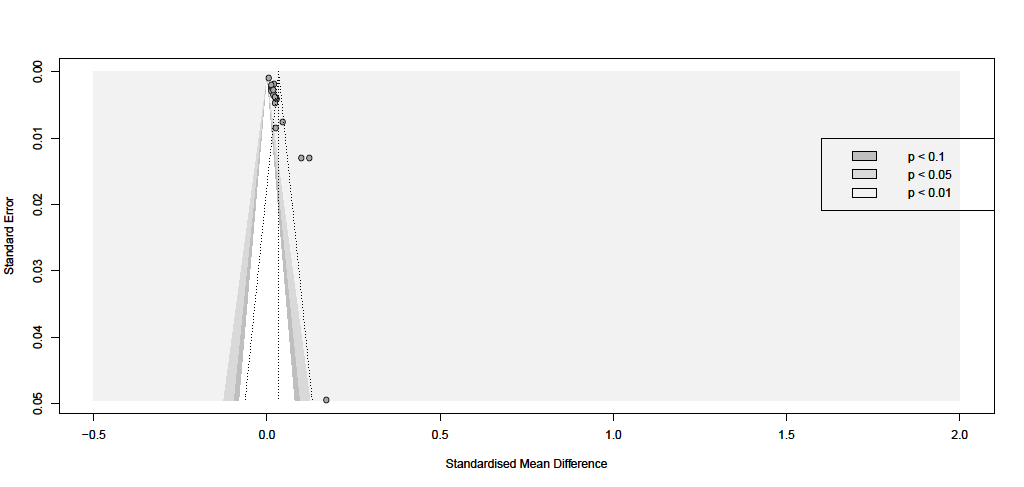


Figure 2: Publication bias of the meta-analysis

**References**

1. Ganesh R, Shrestha D, Bhattachan B, Rai G. Epidemiology of urinary tract infection and antimicrobial resistance in a pediatric hospital in Nepal. BMC Infect Dis. 2019 Dec;19(1):420.

2. Shrestha LB, Baral R, Poudel P, Khanal B. Clinical, etiological and antimicrobial susceptibility profile of pediatric urinary tract infections in a tertiary care hospital of Nepal. BMC Pediatr. 2019 Jan 29;19:36.

3. Raya GB, Dhoubhadel BG, Shrestha D, Raya S, Laghu U, Shah A, et al. Multidrug-resistant and extended-spectrum beta-lactamase-producing uropathogens in children in Bhaktapur, Nepal. Trop Med Health. 2020 Dec;48(1):65.

4. Pyakurel S, Ansari M, Kattel S, Rai G, Shrestha P, Rai KR, et al. Prevalence of carbapenemase-producing Klebsiella pneumoniae at a tertiary care hospital in Kathmandu, Nepal. Trop Med Health. 2021 Dec;49(1):78.

5. Baidya S, Sharma S, Mishra SK, Kattel HP, Parajuli K, Sherchand JB. Biofilm Formation by Pathogens Causing Ventilator-Associated Pneumonia at Intensive Care Units in a Tertiary Care Hospital: An Armor for Refuge. Brul S, editor. BioMed Research International. 2021 May 28;2021:1–10.

6. Koirala S, Khadka S, Sapkota S, Sharma S, Khanal S, Thapa A, et al. Prevalence of CTX-M β-Lactamases Producing Multidrug Resistant Escherichia coli and Klebsiella pneumoniae among Patients Attending Bir Hospital, Nepal. Mehmood K, editor. BioMed Research International. 2021 Jun 8;2021:1–11.

7. Gurung S, Kafle S, Dhungel B, Adhikari N, Thapa Shrestha U, Adhikari B, et al. Detection of OXA-48 Gene in Carbapenem-Resistant Escherichia coli and Klebsiella pneumoniae from Urine Samples. IDR. 2020 Jul;Volume 13:2311–21.

8. Karki D, Dhungel B, Bhandari S, Kunwar A, Joshi PR, Shrestha B, et al. Antibiotic resistance and detection of plasmid mediated colistin resistance mcr-1 gene among Escherichia coli and Klebsiella pneumoniae isolated from clinical samples. Gut Pathog. 2021 Dec;13(1):45.

9. Nepal K, Pant ND, Neupane B, Belbase A, Baidhya R, Shrestha RK, et al. Extended spectrum beta-lactamase and metallo beta-lactamase production among Escherichia coli and Klebsiella pneumoniae isolated from different clinical samples in a tertiary care hospital in Kathmandu, Nepal. Ann Clin Microbiol Antimicrob. 2017 Dec;16(1):62.

10. Shilpakar A, Ansari M, Rai KR, Rai G, Rai SK. Prevalence of multidrug-resistant and extended-spectrum beta-lactamase producing Gram-negative isolates from clinical samples in a tertiary care hospital of Nepal. Trop Med Health. 2021 Dec;49(1):23.

11. Yadav NS, Sharma S, Chaudhary DK, Panthi P, Pokhrel P, Shrestha A, et al. Bacteriological profile of neonatal sepsis and antibiotic susceptibility pattern of isolates admitted at Kanti Children’s Hospital, Kathmandu, Nepal. BMC Res Notes. 2018 Dec;11(1):301.

12. Thapa S, Adhikari N, Shah AK, Lamichhane I, Dhungel B, Shrestha UT, et al. Detection of NDM-1 and VIM Genes in Carbapenem-Resistant Klebsiella pneumoniae Isolates from a Tertiary Health-Care Center in Kathmandu, Nepal. CHE. 2021 Aug 31;1–11.

13. Kayastha K, Dhungel B, Karki S, Adhikari B, Banjara MR, Rijal KR, et al. Extended-Spectrum β-Lactamase-Producing Escherichia coli and Klebsiella Species in Pediatric Patients Visiting International Friendship Children’s Hospital, Kathmandu, Nepal. Infect Dis (Auckl). 2020 Jan 1;13:1178633720909798.

14. Dhungana K, Krishna Awal B, Dhungel B, Sharma S, Raj Banjara M, Raj Rijal K. Detection of Klebsiella pneumoniae Carbapenemase (KPC) and Metallo-Beta Lactamase (MBL) Producing Gram Negative Bacteria Isolated from Different Clinical Samples in A Transplant Center, Kathmandu, Nepal. Act Scie Micro. 2019 Nov 11;2(12):60–9.

15. GC Binod, Sapkota NR, Rayamajhee B, Lamichhane J, Poudel P, Lekhak S, et al. Detection of blaNDM-1 gene among the carbapenem resistant Escherichia coli and Klebsiella pneumoniae isolates from a children’s hospital in Nepal. Novel Research in Microbiology Journal. 2018 Oct 1;2(5):65–74.

16. Kuinkel S, Acharya J, Dhungel B, Adhikari S, Adhikari N, Shrestha UT, et al. Biofilm Formation and Phenotypic Detection of ESBL, MBL, KPC and AmpC Enzymes and Their Coexistence in Klebsiella spp. Isolated at the National Reference Laboratory, Kathmandu, Nepal. Microbiology Research. 2021 Sep;12(3):683–97.
